# Total Mesorectal Excision with New Robotic Platforms: A Scoping Review

**DOI:** 10.3390/jcm13216403

**Published:** 2024-10-25

**Authors:** Francesco Marchegiani, Carlo Alberto Schena, Gaia Santambrogio, Emilio Paolo Emma, Ivan Tsimailo, Nicola de’Angelis

**Affiliations:** 1 Unit of Colorectal and Digestive Surgery, DIGEST Department, Beaujon University Hospital, AP-HP, University of Paris Cité, 92110 Clichy, France; 2 Unit of Robotic and Minimally Invasive Digestive Surgery, Department of Surgery, Ferrara University Hospital, 44124 Ferrara, Italy; 3 Department of Translational Medicine, University of Ferrara, 44121 Ferrara, Italy

**Keywords:** rectal cancer, robotic surgery, robotics, total mesorectal excision, rectal anterior resection, abdominoperineal resection, intersphincteric resection, TaTME

## Abstract

Colorectal surgery is one of the specialties that have significantly benefited from the adoption of robotic technology. Over 20 years since the first robotic rectal resection, the Intuitive Surgical Da Vinci system remains the predominant platform. The introduction of new robotic systems into the market has enabled the first documented total mesorectal excision (TME) using alternative platforms. This scoping review aimed to assess the role and adoption of these emerging robotic systems in performing TME for rectal cancer surgery. **Methods:** A comprehensive search of the Medline, Embase, and Cochrane databases was conducted up to August 2024, following the Preferred Reporting Items for Systematic Reviews and Meta-Analyses extension for Scoping Reviews (PRISMA-ScR) guidelines. **Results:** Thirty-six studies were included in the review. The majority of rectal surgical procedures were performed using eight different robotic platforms. Intraoperative, short-term, and functional outcomes were generally favorable. However, pathological results were frequently incomplete. Several studies identified the lack of advanced robotic instruments as a significant limitation. **Conclusions:** The quality of the resected specimen is critical in rectal cancer surgery. Although TME performed with new robotic platforms appears to be feasible and safe, the current body of literature is limited, particularly in the assessment of pathological and long-term survival outcomes.

## 1. Introduction

Total mesorectal excision (TME) for rectal cancer was introduced in 1982 by Professor Bill Heald, with the goal of removing all the adipose tissue surrounding the rectum, which may contain remnants of tumoral cells, even distal to the tumor site [1]. Despite the conceptual simplicity of this assumption, the surgical procedure remains complex and requires precise anatomy recognition, aimed at achieving both circumferential and distal resection margins that are free of tumor, while also avoiding injury to the surrounding pelvic organs. The technique proposed by Heald involves dissection along a “holy plane”, requiring optimal traction and countertraction within a confined space, without disrupting the mesorectal envelope [2]. The quality of rectal resection is a critical determinant of surgical resection and is strongly correlated with the oncological outcomes [3].

Laparoscopic TME was introduced in the 1990s as the first minimally invasive procedure for rectal cancer treatment [4]. The adoption of this technique faced challenges and was widely debated, as laparoscopy struggled to demonstrate non-inferiority to open surgery [5,6,7,8,9,10]. While the proven benefits of laparoscopy, such as faster recovery, were acknowledged, they had to be weighed against its limitations, including instrument rigidity, two-dimensional visualization, and a steep learning curve [11]. Robotic surgery was introduced 23 years ago and has progressively revolutionized the field of minimally invasive surgery, addressing many of the limitations associated with laparoscopy [12]. Colorectal surgery, particularly rectal surgery, was one of the first specialties to benefit from this technology [13]. The limited operative space in the narrow pelvis was a key reason for the adoption of robotics, by both urologists and colorectal surgeons, with the first documented rectal resection performed in 2003 [14]. The postulated benefits of robotic TME include three-dimensional visualization, high magnification, articulated instruments that mimic the movements of the human hand, tremor filtration, and improved ergonomics [15]. Few studies have directly compared the laparoscopic and robotic approaches for TME. The ROLARR trial failed to demonstrate the superiority of the robotic approach over laparoscopy, but the study had several limitations, primarily related to the training of the participating surgeons [16]. More recently, the REAL trial showed that short-term outcomes after robotic TME were superior to those of laparoscopic TME when performed by experienced surgeons [17]. However, the presented results were the secondary outcome of the study. Additionally, a recent multicenter European propensity score-matched study indicated the superiority of robotic rectal resection over laparoscopy for cancers located in the lower rectum [18].

For more than 20 years, the Intuitive Surgical Da Vinci system has been the only robotic platform available on the market. However, there is a growing number of reports on the role of new robotic platforms in rectal cancer surgery [19].

The Intuitive Surgical Da Vinci^®^ Xi™ (Intuitive Surgical, Sunnyvale, CA, USA) remains the most widely used robotic platform globally, though a fifth-generation model (Intuitive Surgical Da Vinci^®^ 5, Intuitive Surgical, Sunnyvale, CA, USA) has been recently released. Despite significant advancements over previous versions, the current robotic system still has several limitations: the bulky robotic cart that occupies the operative space, the fixed trocar positioning that can restrict multi-quadrant procedures, the lack of haptic feedback, the non-optimal ergonomics, and high operating costs [20,21,22]. In response to these limitations, several competitors have developed new robotic architectures and surgical concepts, some of which are inspired by the Da Vinci system [19].

Given the limited and sometimes conflicting evidence regarding the benefits of robotic surgery in rectal cancer treatment, the introduction of new robotic platforms presents further challenges in evaluating outcomes. This scoping review aimed to evaluate the role and adoption of these new surgical robotic systems in performing total mesorectal excision for rectal cancer surgery, in order to identify and map the current evidence on the pathological, functional, and technical outcomes associated with these platforms and provide clinicians with future research priorities.

## 2. Materials and Methods

### 2.1. Search Strategy and Data Sources

This scoping review was conducted with a systematic approach, adhering to the Preferred Reporting Items for Systematic Reviews and Meta-Analyses extension for Scoping Reviews (PRISMA-ScR) statement [23], to answer the following question: “What is the current evidence supporting the performance of TME with new robotic platforms?”

The first search was conducted in PubMed to identify all the emerging surgical robots currently available or undergoing testing in preclinical and clinical trials. The existing multiport platforms representing the 4 generations of Intuitive Surgical Da Vinci robot (namely, Classic IS1000, S^®^/Si^®^/Xi^®^/X^®^) by Intuitive Surgical, Sunnyvale, CA, USA, were excluded from the review.

The Medline (via PubMed), Embase, and Cochrane Library databases were searched to retrieve studies describing the adoption of new robotic platforms in rectal cancer surgery, up to 20 August 2024. Multiple research queries were formulated using a combination of keywords and MeSH terms, for each database. The search terms included the following keywords: rectal cancer; total mesorectal excision; proctectomy; anterior resection; rectal resection; abdominoperineal resection; intersphincteric resection; Hugo robot; Versius robot; Da Vinci single-port robot; Senhance robot; revo-i robot; Micro Hand robot; Hinotori robot; avatera robot; distalmotion robot; maestro robot; mira robot; enos robot; mantra robot; bitrack system robot; carina robot; vicarious robot; Shurui robot; sp1000 robot; mp1000 robot; toumai robot; surgical robot 01; saroa robot. Multiple spellings of each term were used, combined with the manufacturer’s name or brand, to avoid missing any data due to the use of different nomenclature (e.g., prototype codes or previous model names).

According to the PCC format, the following items were used to select the retrieved articles: P, population: patients > 18 years, affected by primary or recurrent rectal adenocarcinoma, requiring a surgical resection. All types of rectal cancer surgery with TME, including any type of sphincter preservation, resection, or amputation were considered with the exclusion of partial mesorectal excision. C, concept: robotic surgery performed with a surgical platform different from the Intuitive Surgical multiport Da Vinci S^®^/Si^®^/Xi^®^/X^®^. C, context: both tertiary hospital and university hospital settings where these emerging robotic systems have been developed and adopted.

All reported outcomes in the individual studies were considered, including intraoperative, postoperative, pathological exam, short-term postoperative outcomes, functional outcomes, and cost analysis. Moreover, all types of study design were considered, including case reports. Review articles were excluded. If a single case report or a small series was included in a larger series, the study was not included. Where the redundancy of a study could not be assessed (number registration protocol not available or different inclusion period), the study was included and highlighted in the results. Abstracts without a complete case presentation and congress communications were excluded. Only studies in the English language were considered. The literature search and selection were performed by two independent reviewers (F.M., C.A.S.). According to the PRISMA methodology, all records were first merged into a single database, duplicates were removed, and the remaining articles were screened for relevance based on the title and abstract. Disagreements were resolved through discussion and consensus; if no consensus was reached, a senior author was consulted (N.d.A.) in assessing study inclusion. The two reviewers, supported by three additional reviewers (G.S., E.E., I.T.) independently read the full-text articles to confirm their inclusion.

### 2.2. Data Extraction and Synthesis

An electronic spreadsheet was filled with data extracted from the selected studies. The following items were collected: first author’s name, year of publication, type of study design, time frame of the study, robotic platform adopted, total number of patients/procedures, number of patients treated for rectal cancer, patient’s sex, patient’s body mass index (BMI), patient’s age, tumor distance from the anal verge, pre-treatment cTNM, preoperative treatment, type of preoperative treatment, post-treatment cTNM, type of surgical intervention, number of robotic and assistant arms adopted, intraoperative surgical outcomes, postoperative surgical outcomes, pathological outcomes, short-term outcomes, long-term outcomes, functional outcomes, learning curve, and cost analysis.

### 2.3. Quality Assessment

The quality and risk of bias of the included studies was assessed according to the MINORS scoring system. The MINORS system attributes a score of 0 if the item is not reported, 1 if the item is reported but inadequate, or 2 if the item is reported and adequate. The global highest score is 16 for non-comparative studies and 24 for comparative studies. Case reports were not evaluated due to the high risk of bias by definition. Randomized controlled trials were evaluated according to the revised Cochrane risk-of-bias tool for randomized trials (RoB 2) [24].

## 3. Results

**Figure 1:**
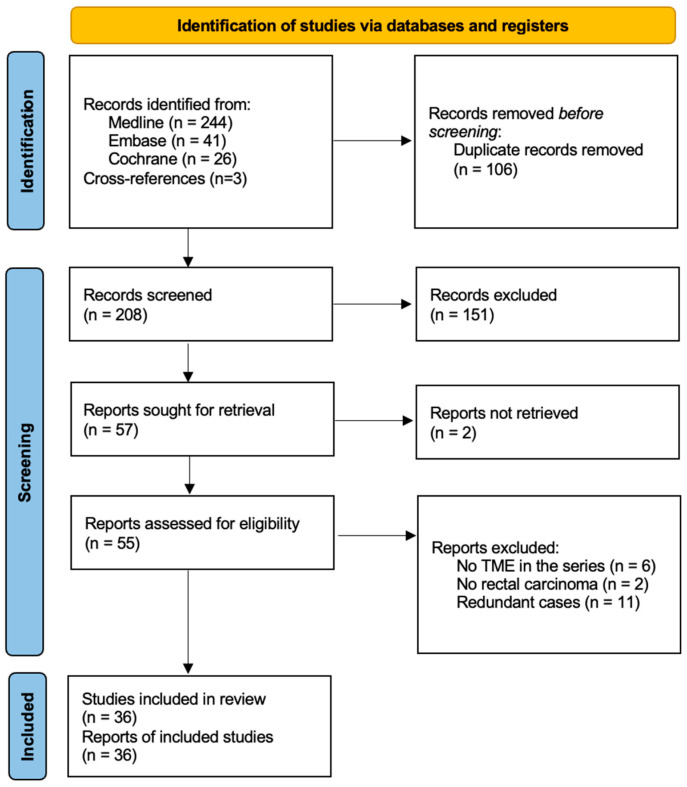
PRISMA flowchart of the literature search and selection.

The initial database search identified a total of 314 studies, of which 106 were duplicates. After screening the titles and abstracts of the 208 remaining articles, 151 were excluded due to the non-relevant specialty or intervention. Two articles could not be retrieved despite an in-depth search that included contacting the corresponding authors. After a full-text review of the 55 eligible articles, an additional 19 were excluded: 11 were redundant cases included in larger and more recent papers, 6 were not related to TME, and 2 were not focused on rectal adenocarcinoma. Ultimately, 36 studies met the inclusion criteria and were selected for the qualitative synthesis of the literature (Figure 1).

Among the included studies, 7 were case reports, 18 were case series, and 9 were comparative studies. Two studies were randomized controlled trials.

Overall, a total of 3896 patients were included, of whom 691 underwent TME with a new robotic platform. Some authors published multiple papers with overlapping inclusion periods. After a thorough analysis of protocol numbers and study periods, it was not possible to determine the exact number of redundant patients.

The most commonly reported procedure was low anterior resection (LAR, *n* = 383), followed by abdominoperineal resection (APR, *n* = 35), intersphincteric resection (ISR, *n* = 27), ultra-low anterior resection (ULAR, *n* = 16), and transanal total mesorectal excision (TaTME, *n* = 6). In 224 cases, the specific surgical procedures were even not reported or combined (Table 1). TaTME cases were hybrid procedures where the robot was used for the abdominal phase. Although the article published by Marks et al. reported on robotic TaTME with a full robotic approach, data extraction was not possible due to mixed statistics [25].

The mean MINORS score was 10 (range: 8–14) for non-comparative studies and 19 (range: 16–20) for comparative studies. One randomized controlled trial (RCT) could not be assessed for risk of bias given that the trial was designed for an outcome (ergonomics) and a comparator (laparoscopy) which are not the object of this review. One RCT presented a high risk of bias.

### 3.1. Robotic Platforms

Eight robotic platforms were used: Asensus Senhance^®^ (Asensus Surgical US, Durham, NC, USA), Medtronic Hugo^™^ RAS (Medtronic, Minneapolis, MN, USA), Intuitive Surgical Da Vinci SP^®^ (Intuitive Surgical, Sunnyvale, CA, USA), Wego Micro Hand S (Shandong WEGO Surgery Robot Co., Ltd., Weihai, China), CMR Versius^®^ (CMR Surgical, Cambridge, UK), Medicaroid Hinotori^™^ (Medicaroid Corporation, Kobe, Hyogo, Japan), KangDuo Surgical Robot-01 (Suzhou KangDuo Robot Co., Ltd., Suzhou, China), and Shurui Endoscopic Surgical Robotic System SR-ENS-600 (Beijing Surgerii Robotics Co. Ltd, Beijing, China) (Table 1).

Three platforms featured a single-cart architecture (Wego Micro Hand S, Medicaroid Hinotori^™^, KangDuo Surgical Robot-01), while three offered a modular design with separate bedside towers (Asensus Senhance^®^, Medtronic Hugo^™^ RAS, CMR Versius^®^). Two of the robotic systems were single-port devices (Intuitive Surgical Da Vinci SP^®^ (Intuitive Surgical, Sunnyvale, CA, USA), Shurui Endoscopic Surgical Robotic System SR-ENS-600).

### 3.2. Baseline Characteristics and Preoperative Treatment

The baseline characteristics of the population are described in Table 2. Comorbidities are not reported by the majority of the studies. Some authors reported Charlson Comorbidity Index [44,52,54,55].

Preoperative treatments are reported by only 18 authors. Protocols largely differed between different countries (Table 2).

### 3.3. Intraoperative Outcomes

Only one intraoperative complication was reported, a bowel serosal tear described by Puntambekar et al., which had no clinical consequences [45].

Operative time (OT) was reported by 26 (72.2%) studies [26,27,28,30,31,32,33,37,39,40,41,42,44,45,46,47,48,49,50,51,52,53,54,55,56,60], whereas nine (25%) studies presented mixed results across different interventions [25,34,35,36,38,43,57,58,59] and one (2.8%) study did not report OT [29] (Table 3). Eight authors provided details of OT including docking time (DT) and console time (CT) [37,39,45,48,49,50,53,60].

Estimated blood loss (EBL) was reported by 22 authors [26,27,28,30,33,37,40,41,44,45,46,47,49,50,51,52,53,54,55,56,57,60]. Six articles presented mixed results [25,34,35,36,38,58] and eight did not report EBL [29,31,32,39,42,43,48,59] (Table 3).

The conversion rate was reported in all the studies, with three of them presenting mixed results for the entire series [25,41,43]. The authors differentiated between conversion to open surgery and conversion to conventional laparoscopy (Table 3). One author set a temporal limit for console time (150 min) after which a planned conversion to laparoscopy was performed to facilitate the learning process without compromising surgical safety [42].

The surgeon’s experience was reported by 22 authors, but only 10 articles referred specifically to colorectal surgery or TME (Table 3).

### 3.4. Short Term Outcomes

Major postoperative complications, classified as Clavien–Dindo grade ≥ III, were reported by 12 authors [34,35,37,41,47,48,51,52,54,55,56,60]. Fifteen articles reported no severe complications [26,27,28,29,30,31,32,33,36,38,39,40,44,49,50]. Four studies presented the data in a mixed format [25,42,43,59] and five did not report these data [45,46,53,57,58] (Table 4).

The anastomotic leak rate was reported in 33 articles [25,26,27,28,29,30,31,32,33,35,37,38,39,40,41,42,43,44,45,46,48,49,50,51,52,53,54,55,56,57,58,59,60], while three did not provide this information [34,36,47] (Table 4).

Length of hospital stay was reported in all studies, though 10 articles presented these data in a mixed format (Table 4) [25,34,35,36,38,42,43,58,59,60].

### 3.5. Energy Devices, Staplers, and Advanced Instruments

All the described robotic platforms, except for the Asensus Senhance^®^ [35] and the Wego Micro Hand S [41,51,53], lack an advanced energy device and the mesorectal dissection was performed using monopolar or bipolar energy.

None of the robotic platforms included a robotic stapling system so laparoscopic stapling was the standard method used by all authors performing a double-stapled anastomosis (Table 3).

Surgical clips were applied by the assistant in most cases, as only the Intuitive Surgical Da Vinci SP^®^ (Intuitive Surgical, Sunnyvale, CA, USA), the Medicaroid Hinotori^™^, and the Asensus Senhance^®^ are equipped with robotic clip appliers.

### 3.6. Pathological Assessment

Pathological results after the analysis of resected specimens were reported in 30 studies [25,26,27,28,29,30,31,32,34,35,37,38,39,40,42,43,44,45,49,50,51,52,53,54,55,56,57,58,59,60]. However, only four studies provided complete data on pathological outcomes, including pathological staging, number of harvested nodes, distal resection margin (DRM), and circumferential resection margin (CRM) with positivity rates and quality of the mesorectum (Table 5) [37,51,55,57].

### 3.7. Functional Outcomes

Functional outcomes were reported by five authors [41,51,52,54,55]. These included evaluations using the International Prostate Symptom Score (IPSS), International Index of Erectile Function (IIEF), and Female Sexual Function Index (FSFI) in two cases [52,56], IPSS and Wexner scores in two other cases [54,55], and IPSS and IIEF in one case [51]. All these evaluations were performed by the same study groups, adopting the Wego Micro Hand S robot, with follow-up reaching one year after surgery [41,51,52,54,55].

### 3.8. Economic Analysis

One article presented an economic evaluation comparing the robotic total mesorectal excision performed with the Wego Micro Hand S and the Intuitive Surgical Da Vinci Si^®^ (Intuitive Surgical, Sunnyvale, CA, USA). The study showed lower total hospital costs (CNY 87,040.1 ± 24,676.9 vs. CNY 125,292.3 ± 17,706.7, *p* < 0.05) and surgery costs (CNY 25,772.3 ± 4117.0 vs. CNY 46,940.9 ± 10,199.7, *p* < 0.05) for the Wego Micro Hand S group [53].

## 4. Discussion

The adoption of robotics for rectal cancer surgery has been rapidly growing despite the limited evidence of its superiority over laparoscopy and TaTME [18,61]. The introduction of new robotic platforms in recent years has been globally welcomed as a driving factor to expand access to robotic technology and reduce its costs [19,62].

This scoping review maps the available evidence on the initial adoption and penetration of these new robotic platforms and their application in rectal cancer surgery.

The literature is extremely recent, being published between 2017 and 2024. Eight different robotic platforms are described. Despite the number of relatively important publications included, several authors have reported redundant studies or single case reports, later followed by larger series, leaving the body of evidence limited. Moreover, some studies have presented overlapping inclusion periods, which increases the risk of duplicate inclusion [51,52,53,54,55]. Another limitation is the publication of generic colorectal series rather than studies specifically focused on rectal cancer treatment. Even when the study quality is high, such as the CMR Versius^®^ data registry (CIT), the clinical and pathological outcomes are often mixed, preventing specific conclusions regarding the quality of rectal resection [47]. While registries are fundamental for the IDEAL development of surgical technology [63,64], the publication by Soumpasis et al. focused primarily on the feasibility and safety without adopting specific indicators of rectal cancer surgery quality [47].

The global quality of the reported studies is extremely reduced and only two randomized controlled trials are included in the present review. The VOLCANO trial was designed to assess the ergonomics of robotic surgery with CMR Versius when compared to laparoscopy. Despite the scope which is far from the evaluation of TME quality, the study was included because of its complete reporting on the outcomes of interest. However, its conclusion is not addressed in the evaluation of the surgical resection. The second RCT compared the KangDuo Surgical Robot-01 to the Da Vinci Xi platform. Despite randomization for the pathologists, which represents a key point in the comparison of the TME quality, the trial included sigmoid cancer, did not offer a proper evaluation of the baseline characteristics of the populations, and did not investigate functional outcomes.

The baseline characteristics of the patients and their comorbidities were reported by only a few authors. Some studies utilized a derived comorbidity index, while the majority reported the American Society of Anesthesiologists (ASA) status, which is useful for assessing the risk of postoperative complications [65].

The intraoperative outcomes are encouraging, with a very low complication rate, documented by a single serosal tear reported [45]. Estimated blood loss (EBL) was generally acceptable, with one author reporting a higher EBL [40] than the proposed cutoff of 250 mL for colorectal surgery [66], whereas registry data revealed ranges of EBL up to 500 mL [47]. Robotic surgery demonstrated the advantage of reducing conversion to open surgery [18,67,68]. Indeed, the conversion rate was low in most studies (2.3–6.9%), with exceptions up to 14.3% [42]. However, case reports often do not include complications, leading to potential bias in reported outcomes [69]. Additionally, some authors performed certain surgical steps laparoscopically to reduce operating times, mitigate arms’ conflict, or avoid conversion [43,45,52,54]. For these reasons, it remains difficult to compare full robotic rectal surgery to hybrid procedures in an early learning phase.

The rate of severe postoperative complications ranged from 0 to 16.7% but comparisons with the existing literature are limited by several factors, such as the low number of included patients and the initial learning curve. For example, the REAL trial documented a Clavien–Dindo ≥ III complication rate of 3% [17] but the surgeons participating in the trial were highly experienced and performed at least 100 laparoscopic or robotic surgeries for colorectal cancer per year with the same robotic device.

A significant limitation of the new robotic platforms in rectal surgery is the reliance on laparoscopic advanced instruments such as staplers, aspirators, clip appliers, and energy devices. One of the key advantages of current robotic systems, such as the Da Vinci multiarm, could be the presence of a wristed stapler which is useful in the narrow pelvis [70,71]. If stapling is performed by the assistant, coordination must be optimal, and if it is performed by the lead surgeon, a de-docking procedure is required, reducing the advantages of robotic access in a confined space. On the other hand, the lack of a robotic stapler could be less critical in cases where an ISR is performed with a manual transanal rectotomy or in cases requiring an APR.

The same disadvantage applies to the absence of advanced bipolar or ultrasonic instruments. However, this is more relevant for vessel sealing than mesorectal dissection. Despite the known risk of thermal radiation damage which makes the ultrasonic devices safer than monopolar electrocoagulation forceps in TME surgery [72], monopolar instruments remain widely used [73]. Recently, solutions such as the double bipolar method [74] have been proposed and applied to the new robotic platforms that lack advanced energy devices [75].

Perhaps, the most critical aspect of rectal surgery, examined in this review, is the quality of the surgical specimen which has a strong correlation with oncological outcomes [76]. Neoadjuvant treatments, often used in rectal cancer, complicate surgical dissection and may reduce its quality, in both open and minimally invasive approaches [77]. Only four studies in this review reported comprehensive pathological assessment, including staging, the number of harvested nodes, DRM length and positivity, CRM positivity, and mesorectal quality [37,51,55,57]. Nevertheless, these outcomes should be considered crucial for evaluating new devices in rectal surgery. The potential advantage of robotic surgery over laparoscopy lies in a reduced CRM positivity rate [78], as recently documented in the REAL [17] and VITRUVIANO trial [79]. In this scoping review, some studies reported a CRM positivity rate higher than the cumulative 5.7% observed in the ROLARR trial [16,34,39,56], despite the small series not being comparable to large, well-designed trials.

One of the most debated limitations in current trials investigating robotic rectal surgery is the assessment of surgeons’ expertise. It remains challenging to establish clear thresholds and parameters that define a surgeon as an “expert” in total mesorectal excision (TME), despite the learning curve being well documented [80]. This review highlights the limited reporting on prior colorectal surgical expertise, which was specifically mentioned in only 10 studies. Many studies are “first-in-human” trials, representing the researchers’ initial experiences with new robotic platforms. Consequently, analyzing and generalizing the outcomes is premature, especially considering that even during the plateau phase of the learning curve, experienced surgeons can continue improving the quality of mesorectal excision [81]. A potential alternative for future trials could be the use of an “expertise-based RCT”, where patients are assigned to surgeons with equivalent levels of expertise [82]. Furthermore, the transferability of robotic skills between different platforms in TME has yet to be fully investigated, as has been explored in other specialties [83].

Regarding long-term functional outcomes, only five studies analyzing the Wego Micro H and S platform reported acceptable anorectal and sexual function outcomes.

## 5. Conclusions

Rectal cancer surgery, more than other specialties, is influenced by the quality of the resected specimen. The use of new robotic platforms for rectal surgery offers a viable alternative to the well-established Intuitive Surgical Da Vinci multiarm system. While rectal resection performed with new robotic devices appeared feasible and safe, being associated with no major complications or mortality, the current literature remains very limited, based exclusively on retrospective studies and lacking the evaluation of specific outcomes for rectal cancer surgical quality (both pathologic and survival outcomes). Thus, we emphasize the need for further larger, high-quality comparative studies that can shed light on the interest and applications of these emerging robotic platforms.

## Figures and Tables

**Table 1:** Surgical procedures and robotic platforms adopted.

References	Country	Robotic Platform	Total (*n* = 3896)	TME (*n* = 691)	Intervention Type	Covering Ileostomy
***Case reports/Technical notes***						
Cheong J.Y. et al. (2022) [26]	Republic of Korea	Intuitive Surgical Da Vinci SP	1	1	1 ISR	1 (100%)
Alshalawi W. et al. (2022) [27]	Republic of Korea	Intuitive Surgical Da Vinci SP	1	1	1 ISR	1 (100%)
Picciariello A. et al. (2023) [28]	Republic of Korea	Intuitive Surgical Da Vinci SP	2	2	2 APR	NA
Mureb A. et al. (2023) [29]	Jordan	CMR Versius	1	1	1 APR	NA
Toyota M. et al. (2024) [30]	Japan	Medtronic Hugo	1	1	1 APR	NA
Miura R. et al. (2023) [31]	Japan	Medicaroid Hinotori	1	1	1 LAR	NR
Ishii M. et al. (2024) [32]	Japan	Medicaroid Hinotori	1	1	1 hTaTME with ISR	1 (100%)
***Case series (prospective and retrospective)***						
Spinelli A. et al. (2017) [33]	Italy	Asensus Senhance	45	12	12 LAR	NR
Lin C-C. et al. (2020) [34]	Taiwan	Asensus Senhance	46	6	3 APR; 2 LAR; 1 TaTME	NR
Samalavicius N. et al. (2022) [35]	Lithuania	Asensus Senhance	57	18	9 LAR; 5 APR; 4 TaTME	NR
Sasaki M. et al. (2022) [36]	Japan	Asensus Senhance	55	8	7 LAR; 1 ISR	NR
Piozzi G.N. et al. (2022) [37]	Republic of Korea	Intuitive Surgical Da Vinci SP	13	6	6 ISR	5 (83.3%)
Marks J.H. et al. (2023) [25]	USA	Intuitive Surgical Da Vinci SP	133	37	MS	MS
Kim H.S. et al. (2023) [38]	Republic of Korea	Intuitive Surgical Da Vinci SP	50	16	16 LAR	4 (25%)
Kim H.J. et al. (2023) [39]	Republic of Korea	Intuitive Surgical Da Vinci SP	11	11	11 ISR	10 (90.9%)
Cho H.J. et al. (2024) [40]	Republic of Korea	Intuitive Surgical Da Vinci SP	10	2	2 ULAR	2 (100%)
Yao Y. et al. (2020) [41]	China	Wego MicroHandS	81	11	NR	MS
Collins D. et al. (2021) [42]	UK	CMR Versius	32	14	NR	4 (28.6%)
Dixon F. et al. (2021) [43]	UK	CMR Versius	160	35	25 LAR; 10 APR	NR
Huscher C. et al. (2021) [44]	Italy	CMR Versius	6	1	1 LAR	1 (100%)
Puntambekar S. et al. (2022) [45]	India	CMR Versius	31	31	21 LAR; 10 ULAR	18 (58.1%)
Wehrmann S. et al. (2022) [46]	Germany	CMR Versius	175	13	13 LAR	NR
Soumpasis I. et al. (2023) [47]	UK	CMR Versius	2083	162	NR	NR
Belyaev O. et al. (2024) [48]	Germany	Medtronic Hugo	70	8	8 LAR	6 (75%)
Caputo D. et al. (2024) [49]	Italy	Medtronic Hugo	3	1	1 LAR	NR
***Comparative studies***						
Alshalawi W. et al. (2023) [50]	Republic of Korea	Intuitive Surgical Da Vinci SP	56	28	7 ISR; 17 LAR; 4 ULAR	NR
Wang Y. et al. (2021) [51]	China	Wego MicroHandS	105	40	38 LAR; 2 APR	22 (57.9%)
Lei Y. et al. (2021) [52]	China	Wego MicroHandS	134	43	42 LAR; 1 APR	33 (78.6%)
Zeng Y. et al. (2021) [53]	China	Wego MicroHandS	28	14	14 LAR	MS
Jiang J. et al. (2021) [54]	China	Wego MicroHandS	90	43	42 LAR; 1 APR	34 (80.9%)
Wang Y. et al. (2022) [55]	China	Wego MicroHandS	99	26	25 LAR; 1 APR	12 (48%)
Liu Y. et al. (2022) [56]	China	Wego MicroHandS	135	43	42 LAR; 1 APR	MS
Guo Z. et al. (2023) [57]	China	Shurui SR-ENS-600	7	3	3 LAR	NR
Noshiro H. et al. (2024) [58]	Japan	Medicaroid Hinotori	63	8	8 LAR	MS
***Randomized trials***						
Dixon F. et al. (2024) [59]	UK	CMR Versius	60	22	14 LAR; 8 APR	NR
Sun Z. et al. (2024) [60]	China	KangDuo Surgical Robot-01	50	21	21 LAR	1 (4.8%)

TME: total mesorectal excision; LAR: low anterior resection; APR: abdominoperineal resection; ISR: intersphincteric resection; ULAR: ultra-low anterior resection; TaTME: transanal total mesorectal excision; hTaTME: hybrid TaTME; NA: not applicable; NR: not reported; MS: mixed statistics.

**Table 2:** Patient demographics and preoperative treatment.

References	Age	Sex	Comorbidities	Preoperative Chemotherapy	Preoperative Radiation
***Case report/Technical notes***					
Cheong J.Y. et al. (2022) [26]	57	F	NR	Yes (100%)	Yes (100%)
Alshalawi W. et al. (2022) [27]	49	M	NR	NR	NR
Picciariello A. et al. (2023) [28]	72 (70–72)	2 F	NR	Yes (100%)	Yes (100%)
Mureb A. et al. (2023) [29]	56	F	No	Yes (100%)	Yes (100%)
Toyota M. et al. (2024) [30]	68	F	NR	NR	NR
Miura R. et al. (2023) [31]	71	Female	NR	No	No
Ishii M. et al. (2024) [32]	55	Male	NR	No	No
***Case series (prospective and retrospective)***					
Spinelli A. et al. (2017) [33]	MS	MS	MS	NR	NR
Lin C-C. et al. (2020) [34]	MS	MS	MS	NR	NR
Samalavicius N. et al. (2022) [35]	MS	MS	NR	13 (76.5%)	13 (76.5%)
Sasaki M. et al. (2022) [36]	MS	MS	MS	NR	NR
Piozzi G.N. et al. (2022) [37]	59.2 (44–82)	3 M; 3 F	MS	Yes (100%)	Yes (100%)
Marks J.H. et al. (2023) [25]	MS	MS	MS	MS	MS
Kim H.S. et al. (2023) [38]	MS	MS	MS	MS	MS
Kim H.J. et al. (2023) [39]	63 (42–71) ^†^	9 M; 2 F	MS	8 (72.7%)	8 (72.7%)
Cho H.J. et al. (2024) [40]	60.5 (50–71)	2 F	MS	1 (50%)	1 (50%)
Yao Y. et al. (2020) [41]	MS	MS	MS	MS	MS
Collins D. et al. (2021) [42]	MS	MS	MS	NR	NR
Dixon F. et al. (2021) [43]	MS	MS	MS	NR	NR
Huscher C. et al. (2021) [44]	MS	MS	MS	No	No
Puntambekar S. et al. (2022) [45]	55.6 (30–75)	45,527	7 diabetes; 9 hypertension; 6 diabetes + hypertension; 1 IHD; 1 DVT	4 (12.9%)	4 (12.9%)
Wehrmann S. et al. (2022) [46]	63 (16) ^#^	MS	NR	NR	NR
Soumpasis I. et al. (2023) [47]	59.0 (±14.3)	94/68	NR	NR	NR
Belyaev O. et al. (2024) [48]	66 (41–85) ^†^	6 M; 2 F	NR	NR	NR
Caputo D. et al. (2024) [49]	72	M	IHD	No	No
***Comparative studies***					
Alshalawi W. et al. (2023) [50]	66.5 (53.7–72.5) ^†^	15 M; 13 F	NR	NR	NR
Wang Y. et al. (2021) [51]	57.4 (±9.5)	20 M; 20 F	NR	Yes in 12.5%	Yes in 12.5%
Lei Y. et al. (2021) [52]	59.51 (±7.7)	24 M; 19 F	NR	23 (53.5%)	23 (53.5%)
Zeng Y. et al. (2021) [53]	61.6 (±11.1)	6 M; 8 F	NR	No	No
Jiang J. et al. (2021) [54]	58.6 (±7.8)	25 M; 18 F	NR	5 (11.6%)	5 (11.6%)
Wang Y. et al. (2022) [55]	59.1 (±10.3)	14 M; 12 F	NR	2 (7.7%)	2 (7.7%)
Liu Y. et al. (2022) [56]	58.8 (±7.0)	25 M; 18 F	NR	24 (55.8%)	24 (55.8%)
Guo Z. et al. (2023) [57]	68.3 (67–69)	2 M; 1 F	1 hypertension and cerebral infarction	No	No
Noshiro H. et al. (2024) [58]	MS	MS	MS	3 (37.5%)	3 (37.5%)
***Randomized trials***					
Dixon F. et al. (2024) [59]	MS	MS	NR	NR	NR
Sun Z. et al. (2024) [60]	MS	MS	MS	NR	NR

NR: not reported; IHD: ischemic heart disease; DVT: deep vein thrombosis. All the reported values are absolute, mean (range) or mean (±SD) if not specified. ^†^ median (range). ^#^ median (IQR).

**Table 3:** Intraoperative data.

References	OT (min)	DT (min)	CT (min)	Rectal Transection	EBL	Conversion (Laparoscopy)	Conversion (Open)	Surgeon’s Experience
***Case report/Technical notes***								
Cheong J.Y. et al. (2022) [26]	180	7	NR	Manual	50	No	No	NR
Alshalawi W. et al. (2022) [27]	185	NR	NR	Manual	30	No	No	NR
Picciariello A. et al. (2023) [28]	180/150	4.66/3.66	NR	NA	25/10	No	No	NR
Mureb A. et al. (2023) [29]	NR	NR	NR	NA	NR	No	No	NR
Toyota M. et al. (2024) [30]	450	NR	163	NA	50	No	No	One surgeon received prescribed training
Miura R. et al. (2023) [31]	262	NR	134	LS	NR	No	No	One surgeon: extensive experience in robotic surgery; Medicaroid specific training; certification by the JSES
Ishii M. et al. (2024) [32]	232	NR	88	Manual	NR	No	No	Cadaver training, under the approval of the JSES

Spinelli A. et al. (2017) [33]	359 (274–501)	13 (2–30)	NR	NR	<50	No	No	Single expert colorectal surgeon
Lin C-C. et al. (2020) [34]	MS	MS	MS	LS	MS	No	No	Four surgeons completed Senhance Surgery Robotic System training
Samalavicius N. et al. (2022) [35]	MS	MS	MS	LS	MS	No	No	NR
Sasaki M. et al. (2022) [36]	MS	MS	MS	NR	MS	No	No	NR
Piozzi G.N. et al. (2022) [37]	280 (240–370) ^#^	7 (5–8) ^#^	157 (112–175) ^#^	Manual	<50	No	No	Expert in robotic multiport rectal surgery and in anus-sparing procedures
Marks J.H. et al. (2023) [25]	MS	MS	MS	NR	MS	MS	MS	NR
Kim H.S. et al. (2023) [38]	MS	MS	MS	LS	MS	No	No	Experienced colorectal surgeon
Kim H.J. et al. (2023) [39]	210 (NR) ^†^	3.17 (NR)	120 (NR)	Manual	NR	No	No	NR
Cho H.J. et al. (2024) [40]	312/275	NR	NR	LS	350/275	No	No	NR
Yao Y. et al. (2020) [41]	279.6(±53.8)	25.2 ± 13.9	NR	NR	116.4 ± 62.8	MS	MS	At least 1 year of robotic surgery experience
Collins D. et al. (2021) [42]	319 (222–408)	NR	204 (85–242)	LS	NR	1 (7.1%)	2 (14.3%)	Three surgeons: extensive laparoscopic colorectal experience, simulation (14 h 45 min), dry lab, and cadaveric training (32 h). One surgeon had a fellowship in robotic surgery
Dixon F. et al. (2021) [43]	MS	MS	MS	NR	NR	MS	MS	8 surgeons: experienced laparoscopic surgeons. One surgeon had robotic surgical training
Huscher C. et al. (2021) [44]	250	NR	NR	LS	0–100	No	No	>1500 surgeries with multiple robots
Puntambekar S. et al. (2022) [45]	124 (NR)	17 (14–25)	51 (43–80)	LS	55 (40–85)	No	No	15 years’ TME experience; 3 years’ Da Vinci experience; cadaveric training
Wehrmann S. et al. (2022) [46]	214 (84) ^#^	NR	NR	LS	100 (12) ^#^	No	No	CMR validated training
Soumpasis I. et al. (2023) [47]	307.1 (±120.7)	NR	NR	NR	<500 mL	8 (5%)	11 (6.9%)	NR
Belyaev O. et al. (2024) [48]	325 (290–420)	12.3	233 (120–332)	NR	NR	No	No	NR
Caputo D. et al. (2024) [49]	435	8	397	LS	150	No	No	One surgeon with large experience in laparoscopic colorectal surgery and Da Vinci surgery
***Comparative studies***								
Alshalawi W. et al. (2023) [50]	184 (150.8–195) ^†^	4 (3–4.75)	73 (60.25–90)	NR	20 (20–50)	No	No	NR
Wang Y. et al. (2021) [51]	339.3(±45.3)	NR	NR	LS	140.9 (±68.5)	No	1 (2.5%)	300 open colorectal surgeries; 100 laparoscopic colorectal surgeries; training in robotic surgery; simple surgeries with the Micro Hand S robot
Lei Y. et al. (2021) [52]	235.03 (235–300)	20.19 (NR)	NR	NR	66.54 (25–200)	No	1 (2.3%)	200 laparoscopic TME; 30 Da Vinci TME; 50 Micro Hand S TME
Zeng Y. et al. (2021) [53]	260.6 (±45.4)	24.2 (±9.4)	143.29 (±36)	LS	123.6 (±60.2)	No	No	Two surgeons: 15 years of laparoscopic experience; 5 years of Da Vinci surgery; 100 Micro Hand S surgeries
Jiang J. et al. (2021) [54]	250 (220–350) ^†^	23 (15–40) ^†^	NR	NR	57 (45–250) ^†^	No	1 (2.3%)	NR
Wang Y. et al. (2022) [55]	295.4 (±38.1)	NR	NR	LS	99.2 (±26.4)	No	1 (2.3%)	One surgeon: open TME since 2012, laparoscopic TME since 2015, robotic TME since 2017. Simple Micro Hand S surgeries
Liu Y. et al. (2022) [56]	235 ± 70.5	NR	NR	NR	66.6 ± 35.2	No	1 (2.3%)	One surgeon: 200 laparoscopic TME, 30 Da Vinci TME, 35 Micro Hand S TME
Guo Z. et al. (2023) [57]	MS	MS	NR	LS	17.66 (10–25)	No	No	NR
Noshiro H. et al. (2024) [58]	MS	MS	MS	LS/Manual	MS	No	No	Three surgeons: sufficient Da Vinci experience; proctoring licenses for Hinotori and Da Vinci. Certified assistants
***Randomized trials***								
Dixon F. et al. (2024) [59]	MS	NR	NR	NR	NR	No	No	20 cases before the trial
Sun Z. et al. (2024) [60]	170 (131.5–195.0) ^#^	4 (4.0–5.5) ^#^	62.5 (50.1–74.6) ^#^	LS	50 (20–50) ^#^	No	No	Three experienced surgeons: >100 Da Vinci surgery procedures; animal training

OT: operative time; DT: docking time; CT: console time; EBL: estimated blood loss; MS: mixed statistics; LS: laparoscopic stapler; NR: not reported; NA: not applicable; JSES: Japan Society for Endoscopic Surgery. All the reported values are absolute values, the mean (range), or the mean (±SD) if not specified. ^†^ median (range). ^#^ median (IQR).

**Table 4:** Postoperative data.

References	CD ≥ III (Rate)	AL (Rate)	LOS
***Case report/Technical notes***			
Cheong J.Y. et al. (2022) [26]	0	0	6
Alshalawi W. et al. (2022) [27]	0	0	6
Picciariello A. et al. (2023) [28]	0	NA	5 / 5
Mureb A. et al. (2023) [29]	0	NA	4
Toyota M. et al. (2024) [30]	0	NA	13
Miura R. et al. (2023) [31]	0	0	10
Ishii M. et al. (2024) [32]	0	0	11
***Case series (prospective and retrospective)***			
Spinelli A. et al. (2017) [33]	0	0	6 (3–19)
Lin C-C. et al. (2020) [34]	1 IIIb (16.7%)	NR	MS
Samalavicius N. et al. (2022) [35]	1 IIIb (5.5%)	1 (7.7%)	MS
Sasaki M. et al. (2022) [36]	0	NR	MS
Piozzi G.N. et al. (2022) [37]	1 IIIa (16.7%)	0	7 (6–10)^#^
Marks J.H. et al. (2023) [25]	MS	MS	MS
Kim H.S. et al. (2023) [38]	0	0	MS
Kim H.J. et al. (2023) [39]	0	0	6 (NR)^†^
Cho H.J. et al. (2024) [40]	0	0	5/8
Yao Y. et al. (2020) [41]	1 IIIa (9.1%)	MS	12.8 (±6.4)
Collins D. et al. (2021) [42]	MS	MS	MS
Dixon F. et al. (2021) [43]	MS	MS	MS
Huscher C. et al. (2021) [44]	0	0	7
Puntambekar S. et al. (2022) [45]	NR	2 (6.4%)	6 (5–12)
Wehrmann S. et al. (2022) [46]	NR	0	9 (6) ^#^
Soumpasis I. et al. (2023) [47]	4 IIIb (2.6%)	NR	7.1 ± 5.2
Belyaev O. et al. (2024) [48]	1 IIIa (12.5%)	1 (12.5%)	10 (7–34) ^†^
Caputo D. et al. (2024) [49]	0	0	7
***Comparative studies***			
Alshalawi W. et al. (2023) [50]	0	0	5 (4–6) ^†^
Wang Y. et al. (2021) [51]	1 IIIa, 2 IIIb (7.5%)	1 (2.5%)	9.2(±3.8)
Lei Y. et al. (2021) [52]	1 IIIb (2.3%)	1 (2.3%)	7.45 (±1.23)
Zeng Y. et al. (2021) [53]	NR	0	13.2 (±6.3)
Jiang J. et al. (2021) [54]	4 IIIa (9.3%)	2 (4.7%)	7 (7–12) ^†^
Wang Y. et al. (2022) [55]	2 IIIa (7.7%)	0	8.9 (±2.8)
Liu Y. et al. (2022) [56]	3 NS (7%)	1 (2.3%)	7.5 (±1.2)
Guo Z. et al. (2023) [57]	NR	0	8 (7–10)
Noshiro H. et al. (2024) [58]	NR	0	MS
***Randomized trials***			
Dixon F. et al. (2024) [59]	MS	MS	MS
Sun Z. et al. (2024) [60]	1 (4.8%)	0	MS

CD: Clavien–Dindo; AL: anastomotic leak; LOS: length of hospital stay; MS: mixed statistics. NR: not reported; NA: not applicable. All the reported values are absolute values, the mean (range), or the mean (±SD) if not specified. ^†^ median (range). ^#^ median (IQR).

**Table 5:** Pathological outcomes.

References	pTNM	Nodes Yield	DRM Length (cm)	DRM Positivity	CRM Positivity	TME Quality
***Case report/Technical notes***						
Cheong J.Y. et al. (2022) [26]	ypT2N0M0	20	NR	0	0	C
Alshalawi W. et al. (2022) [27]	ypT3N1M0	NR	NR	NR	NR	NR
Picciariello A. et al. (2023) [28]	ypT3N0/ypT4bN0	13/18	NR	0	0	C/C
Mureb A. et al. (2023) [29]	ypT3N0	NR	NR	NR	NR	
Toyota M. et al. (2024) [30]	T3N0M0	NR	3	NR	0	C
Miura R. et al. (2023) [31]	T3N0M0	NR	NR	0	0	NR
Ishii M. et al. (2024) [32]	T3N0M0	NR	NR	0	0	NR
***Case series (prospective and retrospective)***						
Spinelli A. et al. (2017) [33]	NR	NR	NR	NR	NR	NR
Lin C-C. et al. (2020) [34]	NR	NR	NR	NR	33%	NR
Samalavicius N. et al. (2022) [35]	NR	NR	3.3 ± 1.7	NR	0	C: 84.6% NC: 15.4% *
Sasaki M. et al. (2022) [36]	NR	NR	NR	NR	NR	NR
Piozzi G.N. et al. (2022) [37]	ypT0 (28.6%) T2 (14.3%) T3 (57.1%) N0 (85.7%) N2a (14.3%)	21 (17–25) ^#^	1.5 (0.9–2.4) ^#^	0	0	C: 83.3% NC: 16.7%
Marks J.H. et al. (2023) [25]	MS	24 (11–58)	MS	0	0	C: 100%
Kim H.S. et al. (2023) [38]	MS	NR	NR	NR	0	C: 100%
Kim H.J. et al. (2023) [39]	ypTxN0 (9.1%) I (36.4%) IIA (27.3%) IIIA (9.1%) IIIB (18.2%)	22 ^†^	1 (0.5–2) ^†^	0	1 (9.1%)	NR
Cho H.J. et al. (2024) [40]	I-IIIA	NR	NR	NR	NR	NR
Yao Y. et al. (2020) [41]	NR	NR	NR	NR	NR	NR
Collins D. et al. (2021) [42]	MS	MS	NR	NR	NR	C: 100%
Dixon F. et al. (2021) [43]	NR	MS	NR	NR	NR	NR
Huscher C. et al. (2021) [44]	pT3N1aM1a	13	NR	NR	NR	C: 100%
Puntambekar S. et al. (2022) [45]	I (25.8%) IIa (29%) IIb (6.4%) IIIa (29%) IIIb (9.7%)	19 (11–36)	NR	0	0	C: 87% NC: 13%
Wehrmann S. et al. (2022) [46]	NR	NR	NR	NR	NR	NR
Soumpasis I. et al. (2023) [47]	NR	NR	NR	NR	NR	NR
Belyaev O. et al. (2024) [48]	NR	NR	NR	NR	NR	NR
Caputo D. et al. (2024) [49]	T1N0	29	NR	NR	NR	C
***Comparative studies***						
Alshalawi W. et al. (2023) [50]	ypT1/T2 (50%) T3/T4a (50%) N0 (75%) N1 (8.92%) N2 (3.57%)	17 (13.25–19) ^†^	2.3 (0.5–5.2) ^†^	0	1 (3.57%)	NR
Wang Y. et al. (2021) [51]	I (10%) II (27.5%) III (62.5%)	15.3 (±4.9)	2.4 (±1.3)	0	2 (5%)	C: 77.5% NC: 15% I: 7.5%
Lei Y. et al. (2021) [52]	NR	17.32 (±4.06)	2.53 (±0.538)	1	1 (2.3%)	C: 81% NC: 12.5% I: 5%
Zeng Y. et al. (2021) [53]	NR	15.8 (±3)	2.3 (±1.1)	0	0	C: 100%
Jiang J. et al. (2021) [54]	NR	16.8 (±1.7)	2.5 (±0.6)	2 (4.7%)	2 (4.7%)	C: 79.1% NC: 11.6% I: 9.3%
Wang Y. et al. (2022) [55]	pT1 (26.9%) pT2 (19.2%) pT3 (38.5%) pT4 (15.4%) pN0 (57.7%) pN1 (42.3%)	15.5 (±5.2)	2.1 (±0.8)	0	1 (3.8%)	C: 84.6% NC: 11.5% I: 3.8%
Liu Y. et al. (2022) [56]	NR	NR	NR	NR	3 (6.9%)	C: 83.3% NC: 9.5% I: 7.1%
Guo Z. et al. (2023) [57]	pT4aN1a (33.3%) pT1N (33.3%) pT1N1a (33.3%)	8 (4–13)	2.16 (2–2.5)	0	0	C: 100%
Noshiro H. et al. (2024) [58]	MS	MS	NR	NR	NR	NR
***Randomized trials***						
Dixon F. et al. (2024) [59]	NR	MS	NR	NR	NR	NR
Sun Z. et al. (2024) [60]	MS	15 (13–19) ^#^	3 (2.3–3.5) ^#^	0	0	NR

DRM: distal resection margin; CRM: circumferential resection margin; TME: total mesorectal excision; NR: not reported; C: complete; NC: nearly complete, I: incomplete; MS: mixed statistics. All the reported values are absolute values, the mean (range), or the mean (±SD) if not specified. ^†^ median (range). ^#^ median (IQR).

## Data Availability

No new data were created or analyzed in this study. Data sharing is not applicable to this article.
